# Troubles socio-émotionnels de l’enfant en milieu Konzo, un syndrome paralytique de nature épidémique associé à une intoxication cyanhydrique d’origine alimentaire en Afrique sub-saharienne

**DOI:** 10.11604/pamj.2018.31.118.11640

**Published:** 2018-10-17

**Authors:** Daniel Okitundu Luwa E-Andjafono, Marie-Therese Sombo Safi Ayanne, Guy Bumoko Makila-Mabe, Jean-Pierre Banea Mayambu, Dieudonné Mumba Ngoyi, Michael Boivin, Jean-Jacques Tamfum-Muyembe, Désiré Tshala-Katumbay

**Affiliations:** 1 Département de Neurologie, Université de Kinshasa, République Démocratique du Congo; 2 Programme National de Nutrition, République Démocratique du Congo; 3 Departement de Médecine Tropicale, Université de Kinshasa, République Démocratique du Congo; 4 Institut National de Recherche Biomédicale, République Démocratique du Congo; 5 Michigan State University, East Lansing, Michigan, USA; 6 Department of Neurology, Oregon Health & Science University, Portland OR, USA

**Keywords:** Cyanure, Konzo, neurocognition, toxicité du manioc, trouble socio-émotionnel, Cyanide, Konzo, neurocognition, cassava toxicity, socioemotional disorder

## Abstract

**Introduction:**

l’objectif de cette étude était d’élucider le profil socio-émotionnel de l’enfant en milieu Konzo, une paralysie toxico-nutritionnelle sévissant en Afrique sub-saharienne.

**Méthodes:**

nous avons évalué le profil socio-émotionnel de 210 enfants dont 123 avec konzo et 87 présumés contrôles sains (4-17 ans d’âge) après interview structuré avec les parents lors d’une enquête épidémio-clinique du konzo en 2011 au Congo-Kinshasa. Le profil neurocognitif était documenté par le KABC-II, le BOT-2 et l’indice global des signes neurologiques du Konzo (IGSNK). Les tests associatifs ont été réalisés par le test de Chi-carré, la régression logistique, dans le cas échéant par modèle linéaire généralisé, au seuil de signification de 0,05.

**Résultats:**

dans l’ensemble, l’irritabilité, la violence physique ou l’inhibition avec ou sans tristesse étaient respectivement retrouvés dans 46,0%, 30,2%, 18,7%; avec un risque accru pour le Konzo (OR = 2,6; IC95%: 1,4 - 4,8; p = 0,001). Le trouble socio-émotionnel était associé à l’insuffisance pondérale (OR: 0,49; IC95%: 0,31 - 0,78; p = 0,002) et à un IGSNK élevé (OR: 1,33; IC 95%: 1,1-1,63; p=0,019); et par ailleurs aggravait les déficits cognitifs dans le Konzo (interaction statut neurologique χ troubles socio-émotionnels, D = 6,297; p = 0,013). Des performances cognitives élevées étaient observées chez les enfants non-Konzo mais avec troubles socio-émotionnels. La concentration moyenne (écart-type ± ET) de thiocyanate urinaire était plus élevé (554,8 ± 371,6 µmol/l) chez les enfants Konzo avec troubles socio-émotionnels.

**Conclusion:**

l’enfant vivant en milieu Konzo présente des troubles socio-émotionnels. Leur nature psychopathologique et l’impact sur la cognition nécessitent des études approfondies.

## Abstract (en)

**Introduction:**

the aim of this study was to describe the socioemotional profile of children living in Konzo-affected areas, an epidemic toxico-nutritional palsy in sub-Saharan Africa.

**Methods:**

we evaluated the socioemotional profile of 210 children, 123 with Konzo and 87 presumed to be healthy (4-17 years) based on a structured interview conducted with their parents during an epidemioclinic survey of Konzo in Congo-Kinshasa in 2011. Neurocognitive profile was identified by the KABC-II, the BOT-2 and the global neurological symptom index of Konzo. Associative tests were carried out by using chi-square test, logistic regression and, where applicable, generalized linear model, at the significance threshold of 0.05.

**Results:**

in general, irritability, physical violence or inhibition with or without sadness were found in 46.0%, 30.2%, 18.7% of children respectively, with an increased risk of Konzo (OR = 2.6; CI95%: 1.4-4.8; p = 0.001). Socioemotional disorder was associated with underweight (OR: 0.49; CI95%: 0.31-0.78; p = 0.002) and with an elevated global neurological symptom index of Konzo (OR: 1.33; CI 95%: 1.1-1.63; p = 0.019); furthermore it exacerbated cognitive impairment in children with Konzo (interaction neurological status-socioemotional disorders D = 6.297; p = 0.013). High cognitive performances were observed in children without Konzo but with socioemotional disorders. The average concentration (standard deviation ± SD) of urinary thiocyanate was higher (554.8 ± 371.6 µmol/l) among children with Konzo associated with socioemotional disorders.

**Conclusion:**

children living in Konzo-affected areas have socioemotional disorders. Their psychopathological status and the effect of Konzo on cognition require in-depth studies.

## Introduction

Le Konzo se présente neurologiquement sous forme d’une paraparésie ou tétraparésie spastique non progressive d’installation brutale, irréversible, avec spasticité d’emblée, possibilité de rémission fonctionnelle partielle et d’épisodes d’aggravation dans une moindre proportion de cas [1-3]. Les personnes vulnérables à la maladie vivent sous régime alimentaire monotone aux produits du manioc amer insuffisamment détoxifié du cyanure, en milieu rural et conditions de vie défavorables. Ces dernières sont caractérisées par une insécurité alimentaire, une malnutrition chronique avec insuffisance d’apport d’acides aminés thiosoufrés [4] et un stress chronique et /ou aigu (pauvreté, sécheresse et conflits armés) [5]. Il s’agit surtout des femmes en âge de procréer, des personnes peu instruites ignorant la toxicité du manioc amer ou des enfants âgés de plus de deux ans en plein développement affectif, social et cognitif, qui sont affectés par la maladie [5, 6]. En plus du déficit de la motricité globale, la positivité du réflexe cheiro-mentonnier comme signe d’atteinte du cortex préfrontal, les troubles visuels permanents et ceux de la motricité fine aux membres supérieurs sans paralysie ont été décrits [7, 8]. Des études récentes ont démontré l’existence des troubles cognitifs et les déficiences en zinc, cuivre et sélénium [9, 10]. Ces conditions dans lesquelles survient le Konzo, sa nature de handicap chronique ainsi que les déficiences nutritionnelles et les troubles cognitifs à lui associés constituent des facteurs de risque cumulatif des troubles socio-émotionnels et comportementaux chez l’enfant en milieu endémique au Konzo. Ces troubles ne sont pas encore décrits. Ainsi, cette étude avait pour objectif exploratoire de décrire les caractéristiques socio-émotionnelles et comportementales telles qu’elles sont perçues par les parents des enfants atteints de Konzo, avec une vue comparative par rapport aux enfants non atteints du même milieu et une analyse de la relation entres les troubles socio-émotionnels et comportementaux, le niveau socio-économique familial, l’état nutritionnel, le statut neurologique et les habiletés neuromotrices et neurocognitives des enfants.

## Méthodes

### Type d’étude

Il s’agissait d’une étude post-hoc sur les données de deux travaux réalisés sur les troubles neuropsychologiques du Konzo chez l’enfant et sur la persistance des épidémies, les aspects phénoménologiques et socio-économiques du Konzo à Kahemba en République Démocratique du Congo [9, 11]. La collecte des données de ces études avait eu lieu du 11 octobre au 18 novembre 2011 et concernait 210 enfants dont 123 affectés par le Konzo et 87 sujets présumés sains ou contrôles.

### Caractéristiques de la population d’étude

Les enfants recrutés appartenaient à des ménages dont les chefs desdits ménages et les parents ont donné leur consentement pour la participation à l’étude. Il s’agissait des ménages dont la taille de la fratrie variait de 1 à 13 avec une moyenne (± écart-type) de 6,14 (± 2,39), sans différence entre les deux groupes d’enfants recrutés. Il n’y avait pas aussi de différence entre les 2 groupes concernant le statut matrimonial de parents et le caractère désirable des naissances. L’âge de ces enfants variait entre 4 ans et 17 ans, sans différence entre les filles et les garçons, les enfants Konzo et contrôles.

Le sex-ratio entre garçons et filles était de 1,12:1 parmi les enfants Konzo et de 1,5:1 chez les contrôles. Les enfants Konzo étaient à différents stades de leur maladie à savoir 92 cas au stade 1 (74,8%); 13 cas au stade 2 (10,6%) et 18 cas au stade 3 (14 ,6%). Il avait été démontré que le risque d’intoxication au cyanure sur la base des taux moyens (± déviation standard) de thiocyanate urinaire mesurés par la méthode semi-quantitative de Howard Bradbury étaient de l’ordre 520,43 (±355,66) micromoles par litre pour les cas et 382,48 (±226,30) micromoles par litre pour les contrôles, des valeurs supérieures au seuil limite d’absence de risque de Konzo de 100 micromoles par litre [11]. Il y avait des différences en défaveur des enfants Konzo pour les indices de la malnutrition chronique comme le retard de croissance (HAZ-WHO) et pour l´insuffisance pondérale (WAZ-WHO) [11]. Les enfants Konzo étaient moins scolarisés que les enfants contrôles présumés sains, 72 sur 123 étaient scolarisés (58,5%) et 51 non scolarisés (41,5%) contre 78 enfants contrôles sur 87 (89,6%) et 9 non scolarisés (10,4%) [11].

### Variables d’intérêt neuropsychologique

Au cours des études ci-haut mentionnées, après anamnèse neuropsychologique, le bilan neuropsychologique a été réalisé par le Bruininks Oseretsky Motor Test of proficiency second édition (BOT-2) et le Kaufman Assessment Battery of Children second édition (K-ABC II) respectivement pour l’évaluation des habiletés neuromotrices et des principaux domaines de compétences neurocognitives [9]. Pour le BOT-2, les scores composites ont été calculés pour le contrôle de la motricité fine, la coordination manuelle et corporelle, l’agilité et la vitesse ainsi qu’un score composite total (TMC). Par le K-ABC-II, la planification, l’apprentissage, l’analyse visuo-spatiale (traitement simultané) et la mémoire de travail (traitement séquentiel) étaient des capacités évaluées pour obtenir le score de l’indice global de fonctionnement cognitif (MPI) [9].

Un indice global de signes neurologiques du Konzo a été calculé pour chaque enfant sur base des résultats de l’examen neurologique concernant les réflexes ostéo-tendineux tricipital, bicipital, rotulien et achilléen, les clonus rotulien et achilléen, le signe de Babinski, le signe d’Oppenheim, le nystagmus, les troubles de la vision, les troubles de l’élocution, le réflexe palmo-mentonnier et les troubles somato-sensitifs. La cote de 0 point correspondait à des réflexes sans particularité, la cote de 1 point à l’hyperréflexie ostéo-tendineuse et au clonus épuisable et la cote de 2 points aux réflexes polycinétiques et au clonus inépuisable. Et pour les autres troubles, l’absence correspondait à la cote 0 et la présence à la cote 2. Le total des points obtenus constituait l’Indice Global de Signes Neurologiques du Konzo (IGSNK ou Total Neurological Signs Score to Konzo exam) [9].

Pour chaque enfant de l’étude, le "Observation for the Measurement of the Environment" (HOME), la version adaptée à l’âge scolaire, a été utilisé pour évaluer les possibilités d’approvisionnement en nourriture, stimulations émotionnelles et cognitives et les possibilités d’apprentissage et la possession des biens matériels de ménages. L’environnement socio-économique familial a été évalué par les 10 domaines du "Observation for the Measurement of the Environment" (HOME), à savoir: la qualité et la quantité de nourriture, l’élevage, l’approvisionnement en eau, les conditions d’hygiène, la qualité physique de la maison, la densité d’occupation et l’équipement matériel de la maison, le niveau de scolarité des parents, la scolarisation des enfants et la bibliothèque familiale [12, 13]. Chaque domaine comprenait plusieurs variables cotées soit none = 0; poor = 1; medium = 2; good = 3 et la somme des cotes correspondait à la cote des domaines partiels du HOME; tandis que la somme des cotes des 10 domaines correspondait à la cote totale de l’environnement socio-économique familial (Home Total), constituant l’indice socio- économique de la famille [11].

Les problèmes socio-émotionnels et de comportement pour les aspects psychopathologiques de la maladie étaient relevés par interview structuré avec le parent de l’enfant pour savoir si l’enfant avait des problèmes connus d’humeur ou d’émotionnalité, sociabilité ou de comportements associés aux émotions et si oui de décrire les émotions et les comportements posant problème. L’analyse des contenus a été faite pour déterminer des problèmes socio-émotionnels et comportementaux des enfants en termes d’irritabilité, agressivité avec violence, inhibition et d’autres réactions.

### Analyse des données

La SPSS version 20.0 a été utilisée pour les analyses statistiques et les productions graphiques. Après une analyse descriptive avec calcul des effectifs et des pourcentages de stéréotypes socio-émotionnels et comportementaux identifiés, les tests de Chi- deux, le modèle linéaire général univarié, les tests non paramétriques de Mann-Whitney et de la médiane ainsi que la régression logistique binaire ont été appliqués selon les besoins analytiques pour établir les liens entre les troubles socio-émotionnels et comportementaux, l’âge, le sexe, l’indice socio-économique, l’état nutritionnel, le statut neurologique et les performances neuropsychologiques, au seuil de signification p = 0,05.

## Résultats

### Caractéristiques socio-émotionnelles et comportementales des enfants Konzo et enfants présumés sains ou contrôles

Les problèmes relatifs à l’humeur, aux émotions et au comportement, rapportés par les parents avaient concerné 139 enfants sur 208 (66,6%) dont 93 enfants konzo contre 46 contrôles. Il n’y a pas eu de source d’information valable dans 2 cas contrôles. Les problèmes émotionnels et de comportement ont été plus rapportés dans le groupe des enfants konzo (75,6% des cas, 93/123) que dans celui des contrôles (54,1% des cas, 46/85), avec une différence statistiquement significative [OR= 2,6; IC95% 1,4 - 4,8; p = 0,001]. Il s’agissait de l’irritabilité excessive dans 46,0% (64/139) dont 37 cas konzo et 27 contrôles), de l’agressivité avec violence physique dans 30,2% (42/139 dont 27 cas konzo et 15 contrôles), de l’inhibition avec ou sans tristesse dans 18,7% (26 /139 dont 22 cas Konzo et 4 contrôles) et de la joie en excès ou de la jovialité sans objet dans 5% (7/139 tous des cas konzo) et de la notion des troubles non rapportés dans 33,2% (69/208 dont 30 cas konzo et 39 contrôles). Le Tableau 1 montre que la fréquence des troubles augmentait en fonction de la sévérité du konzo: 71,7% (66/92) parmi les enfants au stade 1; 84,6% (11/13) chez les enfants au stade 2 et 88,9% (16/18) pour les enfants au stade 3, versus 54,1% (46/85) chez les contrôles. Cette différence de répartition a été trouvée statistiquement significative (Test de Chi-deux d’homogénéité, p = 0,003).

**Tableau 1:** fréquence des problèmes socio-émotionnels et de comportement des enfants Konzo et enfants contrôles ou présumés sains

Statut neurologique	Notion de problème socio-émotionnel et de comportement		
	Non rapportée N (%)	Rapportée N (%)	Total
Stade 1	26 (28,3%)	66 (71,7%)	92 (100%)
Stade 2	2 (15,4%)	11 (84,6%)	13 (100%)
Stade 3	2 (11,1%)	16 (88,9)	18 (100%)
Total	30 (24,3%)	93 (75,7%)	123 (100%)
Contrôles	39 (45,9%)	46 (54,1%)	85 (100%)
Total	69 (33,2%)	139 (66,8%)	208 (100%)

### Facteurs associés aux troubles socio-émotionnels et comportementaux

**Figure 1:**
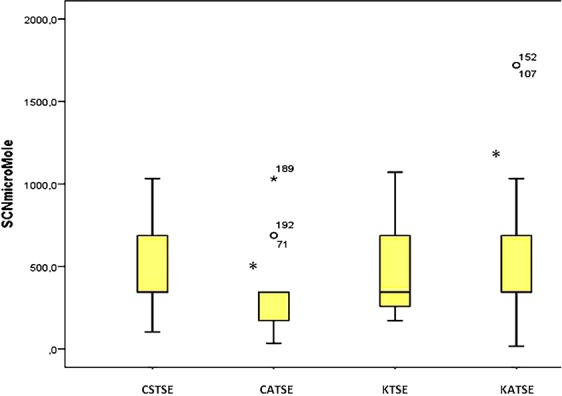
de gauche à droite, SCN micromole: taux urinaires de thiocyanate en micromoles/l; CSTSE = contrôle sans troubles socio-émotionnels; CATSE: contrôle avec troubles socio-émotionnels; KSTSE: Konzo sans troubles socio-émotionnels; KATSE: Konzo avec troubles socio-émotionnels; *p < 0.05 (Bonferroni post-hoc test)

Parmi les enfants déclarés avec troubles socio-émotionnels et comportementaux, il y avait 60,3% (44/73) des garçons contre 39,7% (29/73) des filles (p=0,06), mais parmi les enfants Konzo, la différence de fréquences entre garçons et filles avec troubles n’était pas significative: 53,8% (50/73) contre 46,2% (43 /73) (p = 0,53). Les âges des enfants Konzo avec troubles et sans troubles n’étaient pas significativement différents (p = 0,877, Test de Mann-Whitney). Les enfants atteints et non atteints de Konzo avec troubles socio-émotionnels et comportementaux n’avaient pas des durées de la maladie plus longues ni des indices socio-économiques de la famille (Home Total) plus dégradés. Quant au marqueur de l’intoxication cyanhydrique, les enfants Konzo avec troubles socio-émotionnels avaient des taux urinaires de thiocyanate les plus élevés (Figure 1).

Par rapport aux indices nutritionnels et neurologiques, les enfants atteints de Konzo avec troubles socio-émotionnels et comportementaux étaient plus dégradés que ceux sans troubles de façon statistiquement significative (Test de Mann-Whitney, Tableau 2) en termes d’insuffisance pondérale (WAZ-WHO) (p = 0,001), de malnutrition chronique (HAZ-WHO) (p = 0,044) et de détérioration neurologique (IGSNK) (p = 0,033). Dans un modèle de régression logistique binaire où la probabilité de prédire les troubles affectifs et comportementaux était de 83,3% chez les enfants Konzo, avec une force d’association de 31% (R-Deux de Nagelkerke), l’insuffisance pondérale (OR: 0,49, IC 95% 0,31- 0,78; p = 0,002) et l’indice global des signes neurologiques (OR: 1,33, IC95% 1,1 - 1,63; p = 0,009) étaient les meilleurs prédicteurs de ces troubles (Tableau 3).

**Tableau 2:** liens entre les indices nutritionnels, socioéconomique, familial et de détérioration neurologique, les performances neuromatrices et neurocognitives globales et les troubles socio-émotionnels et comportementaux (Test de Mann-Whitney)

	Troubles affectifs et comportementaux selon l’état neurologique					
Facteurs Associés	Konzo		Non konzo		Tous	
	Oui	Non	Oui	Non	Oui	Non
**SCN (micromoles/l)**						
Moyenne (± ET)	534,8 (± 371,6)	457,8 (± 279,5)	338,7 (± 205,7)	441, 5 (± 230,4)	484,3 (± 346,7)	448,9 (± 250,9)
Médiane (IIQ)	344(344,0)	344,0(473,0)	344,0(172,0)	344,0(344.0)	344,0(344,0)	344,0(344,0)
P	0,52		0 ,089		0,940	
**WAZ-WHO**						
Moyenne (± ET)	-3,161765 (±1,3227097)	-1,954545 (±1,4631106)	-1,700000 (±1,7645943)	-2,125000 (±1,5411035)	-2,714286 (±1,6118121)	-2,043478 (±1,4900637)
Médiane (IIQ)	-3,000000 (2,0000)	-2,000000 (2,0000)	-1,5000 (2,0000)	-2,000000 (2,7500)	-3,000000 (3,0000)	-2,000000 (2,0000)
P	0,001		0,393		0,012	
**HAZ-WHO**						
Moyenne (± ET)	-2,823529 (± 1,3124534)	-2,454545 (± 1,6938021)	-1,733333 (±1,3053890)	- 1,625000 (± 2,1608504)	-2,489796 (± 1,4037964)	-2,021739 (±1,9838572)
Médiane (IIQ)	-3,000000 (1,7500)	-2,000000 (1,2500)	-2,000000 (2,2500)	-2,000000 (2,0000)	-3,000000 (1,0000)	-2,000000 (2,0000)
P	0,044		0,885		0,042	
**IGNK**						
Moyenne (± ET)	11,0 (± 4,34)	8,77 (± 4,32)	1,70(±1,45)	2,71(±1,83)	8,175(±5,99)	5,61(±24,56)
Médiane (IIQ)	11(6)	8,50 (6)	1,00(2)	2,00(3)	9,00(9)	5,00(6)
P	0,033		0, 129		0,002	
					TMC	
Moyenne (± ET)	24,61(± 6,17)	25,60 (± 5,48)	37,09(±7,88)	32,92(±6,58)	28,74(±8,97)	29,74 (±7,09)
Médiane(IIQ)	21, 00 (9,00)	25,00(11,00)	35,50(12,00)	34,00(7,00)	27,00(14,00)	31,00(12,00)
P	0,301		0,045		0,119	
**MPI**						
Moyenne (± ET)	57,86 (± 8,32)	60,53(± 7,41)	63,41(±8,936)	59,36(±9,141)	59,70(±8,89)	59,87(±8,39)
Médiane(IIQ)	56,00 (13,00)	60,00(10,00)	62,00(14,00)	58,00(11,00)	59,00(14,00)	59,00 (11,00)
P	0,107		0, 038		0,924	

Légende : SCN= thiocyanate urinaire ; WAZ-WHO= Indice Poids/Age-Z Score WHO traduisant le statut pondéral; HAZ-WHO= Indice Taille/Age-Z Score WHO indicateur de la malnutrition chronique; IGNK= indice global des signes neurologiques du konzo, un indicateur de la détérioration dans le konzo ; TMC= Total Motor Composite, marqueur de l’habilité neuromotrice global au BOT-2, MPI= Mental Processing Index ou Indice global de fonctionnement cognitif.

**Tableau 3:** prédicteurs nutritionnels et neuropsychologiques des troubles socio-émotionnels et comportementaux dans le Konzo (régression logistique binaire)

Prédicteurs	Coefficient de corrélation partielle	P	OR	IC95%
WAZ-WHO	-0,715	0,002	0,489	0,308 - 0,777
HAZ-WHO	0,213	0,370	1,238	0,776 - 1,974
IGSNK	0,282	0,009	1,325	1,074 - 1,636
T MC	0,082	0,148	1,085	0,971 - 1,213
MPI	-0,019	0,667	0,982	0,902 - 1,068

Légende: WAZ-WHO= Indice Poids/Age-Z Score WHO traduisant le statut pondéral; HAZ-WHO= Indice Taille/Age-Z Score WHO indicateur de la malnutrition chronique; IGNK = indice global des signes neurologiques du Konzo, un indicateur de la détérioration dans le Konzo; TMC = Total Motor Composite, marqueur de l’habilité neuromotrice global au BOT-2, MPI = Mental Processing Index ou Indice global de fonctionnement cognitif

**Figure 2:**
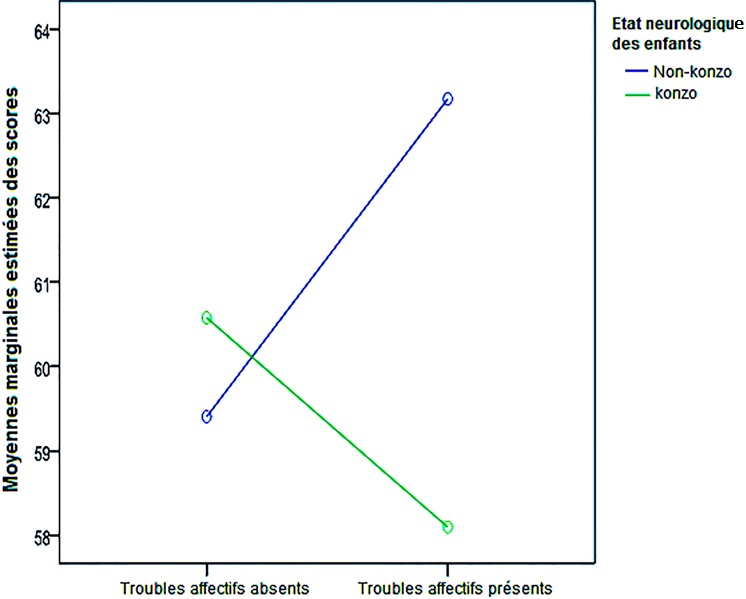
analyse des covariances avec l’indice global de fonctionnement (MPI) comme variable dépendante en ordonnée, le statut Konzo versus non Konzo et les troubles socio-émotionnels et comportementaux (troubles affectifs) comme variables indépendantes, et l’indice de l’environnement socio-économique familial (Home Total) comme covariable, les cas Konzo présentant des troubles socio-émotionnels et comportementaux avaient des performances plus basses de MPI par rapport aux cas Konzo sans troubles et aux non Konzo avec troubles, dans les mêmes conditions d’environnement socio-économique familial

Il n’y avait pas des différences entre les enfants avec et sans troubles pour l’habilité motrice globale (TMC) et l’indice global de fonctionnement cognitif (MPI) et parmi les enfants non atteints de Konzo (Tableau 2). Paradoxalement, les enfants présumés sains avec troubles socio-émotionnels et comportementaux avaient des performances neuromotrices (Test de Mann-Whitney, p = 0,045) et neurocognitives (Test de Mann-Whitney, p = 0,038) plus élevées à celles de ceux de la même catégorie sans troubles (Tableau 2). Par ailleurs, l’interaction entre le statut neurologique Konzo et la présence des problèmes socio-émotionnels et comportementaux déterminaient des faibles performances de fonctionnement cognitif global (MPI) dans les mêmes conditions socioéconomiques familiales (D = 6,29, p = 0,013, Modèle linéaire généralisé univarié) (Figure 2).

## Discussion

Les données de la présente étude rendent compte de l’existence des troubles socio-émotionnels et comportementaux chez l’enfant en milieu Konzo, avec risque accru pour les enfants Konzo (OR = 2,6). Dans les suites des études récentes sur les troubles cognitifs dans le Konzo [9, 14], ce travail ajoute à la clinique neurologique et neuropsychologique du Konzo les troubles socio-émotionnels et comportementaux, complétant ainsi le profil clinique du Konzo. Toutefois, les formes cliniques de ces troubles restent à déterminer par des études ultérieures au moyen des outils de recherche de validité connue comme le "Child Behaviour Check List" ou "Strengths and Difficulties Questionnaire" [15]. Les données de notre étude neurologique montrent que ces troubles peuvent être attribués aux effets de la malnutrition et de la détérioration neurologique. Cependant, il est possible que les troubles observés soient aussi un résultat de réactions psychologiques au handicap moteur ou de déficiences en oligo-éléments comme le zinc, le cuivre et le sélénium déjà documentés dans le Konzo [10, 16, 17].

Bien que sans lien significatif avec le genre, l’âge et la durée de la maladie, les troubles socio-émotionnels et comportementaux observés étaient associés à des performances cognitives plus faibles chez l’enfant Konzo. Cette association avec les faibles capacités cognitives et l’augmentation de leur fréquence avec la sévérité du Konzo suggèrent l’existence d’un syndrome psycho-organique par intoxication cyanhydrique chronique pouvant répondre à un stade avancé à une encéphalopathie chronique. Les taux de thiocyanate urinaire plus élevés en cas de Konzo avec troubles socio-émotionnels et comportementaux suscitent entre autres la question du rôle que jouerait le thiocyanate dans la pathogénie de ces troubles. L’intoxication cyanhydrique non létale provoque l’encéphalopathie [18, 19] et le thiocyanate, dérivé métabolique de l’acide cyanhydrique, est connu comme neurotoxique [20]. Les manifestations neurotoxiques de thiocyanate pourraient être mises en rapport avec une augmentation éventuelle de l’activité du glutamate sur les récepteurs AMPA (α-amino-3-hydroxy-5-methyl-4-isoxazole propionic acid) et NMDA (N-Methyl-D-Aspartate) et comprendraient entre autres la nervosité, l’irritabilité, la confusion, l’excitation maniaque, le délirium, les convulsions et la psychose [21]. A noter que l’activité glutamatergique, par les récepteurs susmentionnés, module la motricité, l’émotion, l’humeur, le stress, l’anxiété et l’agressivité [22]. Les vrais mécanismes à la base des troubles observés restent cependant à établir.

Il est connu dans le milieu Konzo que les enfants atteints ou non de Konzo ont un développement cognitif insuffisant par rapport aux enfants de milieu indemne de Konzo [9], mais la supériorité des enfants présumés sains avec troubles socio-émotionnels et comportementaux en capacités neuromotrices et cognitives par rapport à ceux du même groupe sans troubles et d’autres enfants, au même niveau socio-économique familial, rappelle la problématique des troubles socio-émotionnels et comportementaux prévalant chez des enfants à haut potentiel intellectuel [23-26]. Cette observation mérite une attention particulière dans les études à venir sur le développement social, affectif et cognitif chez l’enfant dans les cinq premières années de la vie en milieu endémique au Konzo caractérisé par des facteurs de stress multiple et cumulatif dont l’intoxication cyanhydrique chronique.

## Conclusion

En conclusion, les troubles socio-émotionnels et comportementaux, ainsi décrits qualitativement pour la première fois dans le Konzo, devraient être désormais comptés parmi les signes cliniques du Konzo chez l’enfant. Ils constituent un marqueur neuropsychiatrique, dont la valeur sémiologique et la psychopathologie seraient différentes chez l’enfant atteint de Konzo et l’enfant présumé sain en milieu Konzo. La nature psychopathologique, les formes cliniques de ces troubles et leur impact sur la cognition nécessitent des études approfondies.

### Etat des connaissances actuelles sur le sujet

- Le Konzo est une maladie neuro-toxico-nutritionnelle associée à l’intoxication cyanhydrique d’origine diététique par la consommation des produits du manioc amer mal détoxifié, qui affecte plus les enfants et la femme en âge de procréer et survient dans des conditions de pauvreté, insécurité alimentaire et malnutrition;
- Ces conditions comportent les risques des troubles cognitifs, socio-émotionnels et comportementaux;
- Les signes cliniques connus du Konzo consistent en des troubles moteurs, les troubles cognitifs venaient d’être décrits mais les troubles socio-émotionnels et comportementaux pas encore.

### Contribution de notre étude à la connaissance

- La présente étude décrit pour la première fois les troubles socio-émotionnels et comportementaux dans le Konzo, complétant ainsi la clinique du Konzo comme maladie neurotoxique;
- Cette étude relance la question de la toxicité nerveuse du thiocyanate dans le Konzo.

## Conflits d’intérêts

Les auteurs ne déclarent aucun conflit d’intérêts.
